# Supplementary material for: COZOID: contact zone identifier for visual analysis of protein-protein interactions
Source: BMC Bioinformatics. 2018 Apr 6;19:125. doi: 10.1186/s12859-018-2113-6 (PMC5889581; doi:10.1186/s12859-018-2113-6)
Supplement: Supplementary file 2 — Software build. Executable binary file of the software tool. (ZIP 118,784 kb) [file 12859_2018_2113_MOESM2_ESM.zip › cozoid/platform/docs/testng-6.5.1-javadoc.zip!/org/testng/xml/package-tree.html]

org.testng.xml Class Hierarchy (TestNG)


---


|  |  |  |
| --- | --- | --- |
| **Overview** \| **Package** \| Class \| **Tree** \| **Deprecated** \| **Index** \| **Help** | |  |
| **PREV**   NEXT | **FRAMES**    **NO FRAMES**     **All Classes** |  |


---


## Hierarchy For Package org.testng.xml

**Package Hierarchies:**: All Packages

---

## Class Hierarchy

- java.lang.Object
  - org.testng.xml.**XmlClass** (implements java.lang.Cloneable, java.io.Serializable)- org.testng.xml.**XmlInclude** (implements java.io.Serializable)- org.testng.xml.**XmlMethodSelector**- org.testng.xml.**XmlPackage** (implements java.io.Serializable)- org.testng.xml.**XmlSuite** (implements java.lang.Cloneable, java.io.Serializable)- org.testng.xml.**XmlTest** (implements java.lang.Cloneable, java.io.Serializable)- org.testng.xml.**XmlUtils**

---


|  |  |  |
| --- | --- | --- |
| **Overview** \| **Package** \| Class \| **Tree** \| **Deprecated** \| **Index** \| **Help** | |  |
| **PREV**   NEXT | **FRAMES**    **NO FRAMES**     **All Classes** |  |


---
